# International proficiency trial for bovine viral diarrhea virus (BVDV) antibody detection: limitations of milk serology

**DOI:** 10.1186/s12917-022-03265-w

**Published:** 2022-05-06

**Authors:** Kerstin Wernike, Martin Beer

**Affiliations:** Institute of Diagnostic Virology, Friedrich-Loeffler-Institut, Südufer 10, 17493 Greifswald – Insel Riems, Germany

**Keywords:** Pestivirus, Bovine viral diarrhea virus, BVDV, Diagnostics, Serology, Monitoring, Milk, Pooled samples, Proficiency testing

## Abstract

**Background:**

Control programs were implemented in several countries against bovine viral diarrhea (BVD), one of the most significant cattle diseases worldwide. Most of the programs rely on serological diagnostics in any phase of the program. For the detection of antibodies against BVD virus (BVDV), neutralization tests as well as a variety of (commercially available) ELISAs are used. Here, test systems applied in various laboratories were evaluated in the context of an international interlaboratory proficiency trial. A panel of standardized samples comprising five sera and five milk samples was sent to veterinary diagnostic laboratories (*n*=51) and test kit manufacturers (*n*=3).

**Results:**

The ring trial sample panel was investigated by nine commercially available antibody ELISAs as well as by neutralization tests against diverse BVDV-1, BVDV-2 and/or border disease virus (BDV) strains. The negative serum and milk sample as well as a serum collected after BVDV-2 infection were mostly correctly tested regardless of the applied test system. A serum sample obtained from an animal immunized with an inactivated BVDV-1 vaccine tested positive by neutralization tests or by total antibody or E^rns^-based ELISAs, while all applied NS3-based ELISAs gave negative results. A further serum, containing antibodies against the ovine BDV, reacted positive in all applied BVDV ELISAs, a differentiation between anti-BDV and anti-BVDV antibodies was only enabled by parallel application of neutralization tests against BVDV and BDV isolates. For the BVDV antibody-positive milk samples (*n*=4), which mimicked prevalences of 20% (*n*=2) or 50% (*n*=2), considerable differences in the number of positive results were observed, which mainly depended on the ELISA kit and the sample incubation protocols used. These 4 milk samples tested negative in 43.6%, 50.9%, 3.6% and 56.4%, respectively, of all investigations. Overall, negative results occurred more often, when a short sample incubation protocol instead of an over-night protocol was applied.

**Conclusions:**

While the seronegative samples were correctly evaluated in most cases, there were considerable differences in the number of correct evaluations for the seropositive samples, most notably when pooled milk samples were tested. Hence, thorough validation and careful selection of ELISA tests are necessary, especially when applied during surveillance programs in BVD-free regions.

## Background

Bovine viral diarrhea (BVD) is one of the most significant cattle diseases worldwide, as it induces major economic losses and represents a substantial issue on animal welfare [1–4]. The causative agent, bovine viral diarrhea virus (BVDV), is a pestivirus (family Flaviviridae), which exists in the two distinct species BVDV-1 (syn. *Pestivirus A*) and BVDV-2 (syn. *Pestivirus B*) [5]. BVDV is closely related to the other two classical pestivirus species border disease virus (BDV, syn. *Pestivirus D*) and classical swine fever virus (CSFV, syn. *Pestivirus C*) [5]. During the last years, further, so-called “atypical” pestiviruses have been described [6–11], among them HoBi-like viruses (syn. BVDV-3 or *Pestivirus H*). Hobi-like viruses were originally isolated from fetal calf serum (FCS), infect cattle and could interfere like e.g. the ovine BDV with BVDV diagnostics because of a genetic and antigenic relatedness [12–15].

The single-stranded positive-sense RNA genome of BVDV encodes four structural proteins, namely the capsid protein C and the envelope glycoproteins E^rns^ (formerly known as E0), E1 and E2, and at least eight non-structural proteins (N^pro^, p7, NS2, NS3, NS4A, NS4B, NS5A and NS5B) [16]. The resulting polyprotein is co- and post-translationally processed by cellular and viral proteases into the individual proteins [17]. The immunodominant proteins for the induction of antibody responses are E^rns^, E2 and the non-structural protein NS3 (also referred to as p80) [18, 19]. Neutralizing antibodies are mainly directed against the glycoprotein E2 [16].

Acutely infected, BVDV-naïve cattle show either none or mild to moderate unspecific clinical signs including diarrhea, fever or pneumonia. However, also severe forms characterized by hemorrhagic syndromes and mucosal disease-like lesions may occur, mainly associated with virulent BVDV-2 strains [20–22]. The clinical consequences of BVDV infection of naïve pregnant cows depend on the phase of gestation and could result in stillbirth, abortion or congenital malformation. When infection occurs during the first three month of pregnancy, it could lead in a high percentage to the birth of persistently infected (PI), immunotolerant, life-long viremic calves [23–25]. As PI animals are unable to develop a specific immunity against the virus strain they are infected with, they shed enormous amounts of BVDV throughout their lives, which makes them the major source for virus perpetuation within individual cattle herds and spread to BVDV-free holdings [26–30].

Due to their crucial role in the spread of BVDV, PI calves are the major target of disease control programs, which are in place in several countries [28, 31–35]. Despite the common goal of virus eradication from the respective cattle population, different approaches were selected for the programs. While the “Scandinavian model” was based on large-scale milk serology, the “Swiss model” was based on the direct antigen or viral genome testing of all animals without serological pre-screening [34, 36]. The latter proved beneficial especially for regions with a high initial virus prevalence and a high level of cattle trading and transport combined with ongoing vaccination campaigns. The centerpiece of the “Swiss approach”, which was also adopted in e.g. Germany and Ireland [31, 37, 38], is the detection of PI animals as early as possible, mainly by ear-notch based testing of every new-born calf for the presence of viral antigen or genome, and their elimination from the respective cattle population. Once all PI animals are removed, non-vaccinated herds become gradually seronegative, allowing for serology-based monitoring of the disease-free status. In those non-vaccinated, BVDV-free herds, bulk milk serology may be used to screen for virus introduction. As an alternative approach, spot-testing of young animals older than 6 months (to avoid the negative influence of maternally derived antibodies acquired by colostrum intake) could be applied [39–43]. The “Scandinavian model” on the other hand, was directly based on large-scale serology to preselect farms with an elevated risk for the presence of PI animals. Thereafter, all animals from herds with high antibody levels were tested individually for virus genome or antigen and the detected PI animals were removed. Finally, an ongoing serological monitoring was established [34]. Hence, in their final phase, both approaches rely on serology-based monitoring of the disease-free status.

For serological diagnostics of previous BVDV infections, neutralization assays represent the gold standard test, as they offer very high sensitivity and specificity. By neutralization tests, antibodies directed against BVDV-1, BVDV-2 and BDV may be differentiated from each other, despite the serological cross-reactivity that exists between these virus species [44]. However, as neutralization tests, which rely on cell-culture systems, are labor-intensive and time-consuming, commercial ELISA tests are used much more frequently during routine diagnostics, as they allow for a more convenient high throughput testing.

During recent studies, varying and sometimes poor sensitivities were observed for BVDV antibody ELISAs [45–47]. Here, test systems used for serological BVDV diagnostics in various laboratories have been evaluated in the context of an international interlaboratory proficiency trial. A panel of standardized and blinded samples was sent to veterinary diagnostic laboratories and test kit manufacturers with the request to analyze the samples by methods routinely applied in the respective institution.

## Results

Five individual sera (sample IDs 01/21 to 05/51) and one individual (09/21) and four pooled milk samples (06/21 to 08/21 and 10/21) were sent to the participants of the proficiency trial (Table 1). This sample panel was investigated by 51 veterinary diagnostic laboratories and 3 kit manufacturers by using nine commercially available and one in-house antibody ELISA. The applied test systems are listed in Table 2. In some cases, several ELISA tests or sample incubation protocols were used, whereby 71 result sets were generated for the sera and 55 for the milk samples. In addition or alternatively to the analysis by antibody ELISA, the sera were tested in 28 laboratories by the cell-culture based standard microneutralization test against diverse BVDV-1, BVDV-2 and/or BDV strains

**Table 1:** Sample panel sent to the participants of the interlaboratory proficiency trial for BVDV serology

sample ID	material	sample status	NT titer (of corresponding sera for milk)
01/21	serum	BVDV antibody-positive, anti-BVDV-2, after infection	Fig. 2, 180 against BVDV-1, 1812 against BVDV-2, 11 against BDV
02/21	serum	BVDV antibody-negative	Fig. 2, < 5 against BVDV-1, BVDV-2 and BDV
03/21	serum	BVDV antibody-positive, anti-BVDV-1, animal immunized with inactivated vaccine	Fig. 2, 572 against BVDV-1, 90 against BVDV-2, 23 against BDV
04/21	serum	BVDV antibody-negative	Fig. 2, < 5 against BVDV-1, BVDV-2 and BDV
05/21	serum	BDV antibody-positive, after infection	Fig. 2, 143 against BVDV-1, 9 against BVDV-2, 720 against BDV
06/21	milk	pooled sample, 4 seropositive animals + 4 negative milk samples	226, 160, 160, 113
07/21	milk	pooled sample, 2 seropositive animals + 8 negative milk samples	1280, 40
08/21	milk	pooled sample, 4 seropositive animals + 4 negative milk samples	57, 40, 28, 14
09/21	milk	BVDV antibody-negative	n.a.
10/21	milk	pooled sample, 2 seropositive animals + 8 negative milk samples	80, 80

Legend: Sample identifier, material and sample status are given. The serum samples 01/21 to 05/21 were tested by the participants by microneutralization tests against BVDV and BDV, the resulting neutralizing (NT) titers from the author’s laboratory are indicated in the table and the titers measured in all participating laboratories are shown in Fig. 2. For the milk samples 06/21 to 10/21, the corresponding sera were tested against BVDV-1 isolate NADL and the resulting titers are given. For the samples 06/21, 07/21, 08/21 and 10/21 only the titers of the seropositive samples are indicated. n.a. – not available

**Table 2:** Results of BVD antibody ELISAs performed by the participating laboratories. The ELISA format (competitive or indirect) is given in brackets following the name and manufacturer of the test.

test system		01/21 (positive, anti-BVDV-2)	02/21 (negative)	03/21 (positive anti-BVDV-1)	04/21 (negative)	05/21 (positive, anti-BDV)	06/21 (positive)	07/21 (positive)	08/21 (positive)	09/21 (negative)	10/21 (positive)
Monoscreen AbELISA BVDV (E0)/blocking, Bio-X Diagnostics S.A. (competitive)	no. tests	2	2	2	2	2	0	0	0	0	0
	no. positive	2	0	2	0	2					
	no. doubtful	0	0	0	0	0					
	no. negative	0	2	0	2	0					
Monoscreen AbELISA BVDV (NS3)/blocking, Bio-X Diagnostics S.A. (competitive)	no. tests	1	1	1	1	1	1	1	1	1	1
	no. positive	1	0	0	0	1	1	0	0	0	0
	no. doubtful	0	0	0	0	0	0	0	0	0	0
	no. negative	0	1	**1**	1	0	0	**1**	**1**	1	**1**
ID Screen® BVD p80 Antibody Competition, Innovative Diagnostics (competitive)	no. tests	27	27	27	27	27	22	22	22	22	22
	no. positive	27	0	0	0	27	21	18	19	**1**	18
	no. doubtful	0	0	0	0	0	0	1	0	0	1
	no. negative	0	27	**27**	27	0	**1**	**3**	**3**	21	**3**
BVDV Total Ab Test, IDEXX (indirect)	no. tests	24	24	24	24	24	16	16	16	16	16
	no. positive	24	0	24	0	24	16*	0	0	0	0
	no. doubtful	0	0	0	0	0	0	0	0	0	0
	no. negative	0	24	0	24	0	0	**16**	**16**	16	**16**
BVDV p80 Ab Test, IDEXX (competitive)	no. tests	1	1	1	1	1	1	1	1	1	1
	no. positive	1	0	0	0	1	0	0	0	0	0
	no. doubtful	0	0	0	0	0	1	0	0	0	0
	no. negative	0	1	**1**	1	0	0	**1**	**1**	1	**1**
Svanovir® BVDV-Ab Screening, SVANOVA (indirect)	no. tests	3	3	3	3	3	3	3	3	3	3
	no. positive	3	0	3	0	3	3	3	1	0	2
	no. doubtful	0	0	0	0	0	0	0	0	0	0
	no. negative	0	3	0	3	0	0	0	**2**	3	**1**
Svanovir® BVDV-Ab Confirmation, SVANOVA (indirect)	no. tests	8	8	8	8	8	9	9	9	9	9
	no. positive	8	0	8	0	8	9	8	3	0	4
	no. doubtful	0	0	0	0	0	0	0	0	0	0
	no. negative	0	8	0	8	0	0	**1**	**6**	9	**5**
PrioCheck^TM^ Ruminant BVD p80 Ab Serum & Milk Kit, Thermo Fisher Scientific (competitive)	no. tests	3	3	3	3	3	2	2	2	2	2
	no. positive	3	0	0	0	3	1	0	0	0	1
	no. doubtful	0	0	0	0	0	0	0	0	0	0
	no. negative	0	3	**3**	3	0	**1**	**2**	**2**	2	**1**
PrioCheck^TM^ Bovine BVDV Ab Plate Kit, Thermo Fisher Scientific (competitive)	no. tests	1	1	1	1	1	1	1	1	1	1
	no. positive	1	0	0	0	1	1	1	1	0	1
	no. doubtful	0	0	0	0	0	0	0	0	0	0
	no. negative	0	1	**1**	1	0	0	0	0	1	0
in-house (unknown)	no. tests	1	1	1	1	1	0	0	0	0	0
	no. positive	1	0	1	0	1					
	no. doubtful	0	0	0	0	0					
	no. negative	0	1	0	1	0					

Legend: The serological sample status is given below the respective sample identifier. Results divergent from the defined sample status are printed in bold. * Five of the 16 participants that used this ELISA initially evaluated the sample “doubtful”, taking the cut-off for individual milk samples as basis (S/*P* < 0.20 negative; 0.20 ≤ S/*P* < 0.30 suspect; S/*P* ≥ 0.30 positive). When applying the cut-off for bulk milk (S/*P *< 0.20 negative; S/P ≥ 0.20 positive), the sample needs to be assessed as “positive”, which was corrected in this table.

**Fig. 1:**
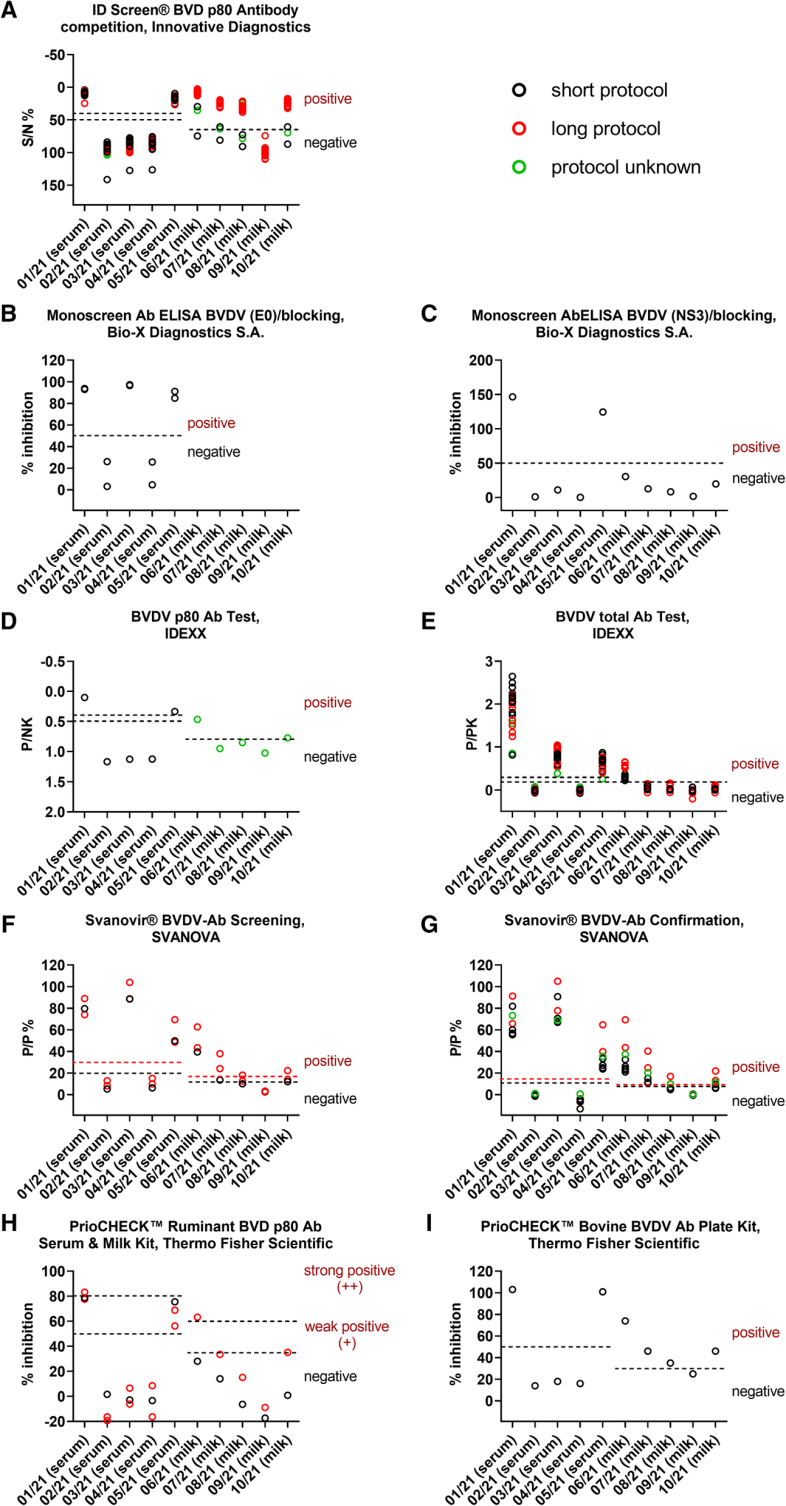
Results of the commercial BVD antibody ELISAs. Results generated using the respective short incubation protocol are shown in black and results produced by the long sample incubation protocol are depicted in red. Green circles represent results for which the participating laboratory did not indicate the applied protocol. The cut-offs are indicated by horizontal dashed lines (black for short protocol, red for long protocol). When the same cut-off is to be used for both protocols, the line is colored in black. A) Three participants indicated their results in the unit PI%, these results were converted into S/N% for the generation of the figure. Two further participants used another, not further specified unit, these results are not shown

The results generated by the commercial ELISAs are shown in Fig. 1 separately for each test kit.

**Fig. 2:**
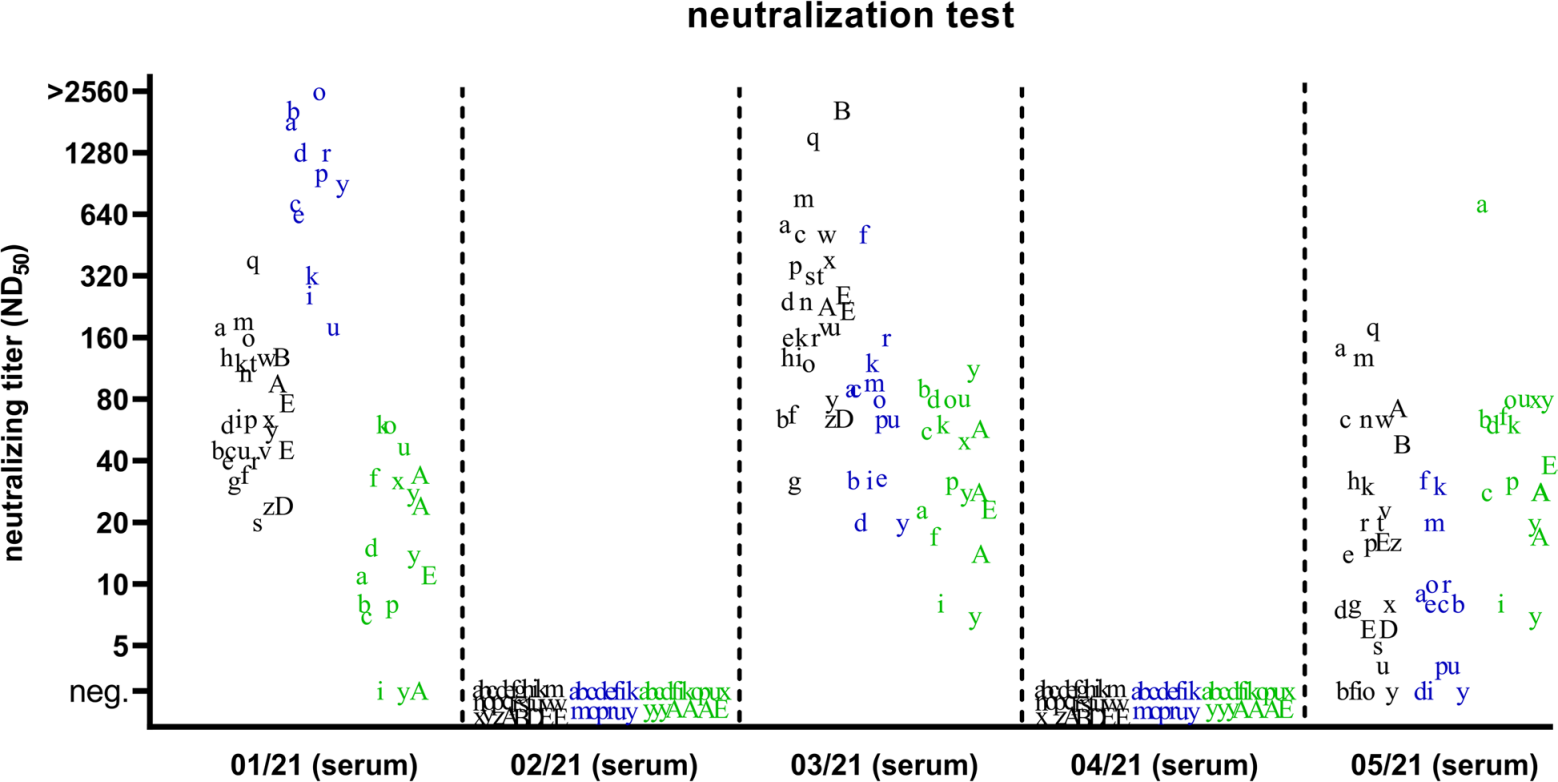
Results of the neutralization tests. The sera were analyzed by the participating laboratories against diverse BVDV-1 isolates (black), against BVDV-2 (blue), or against BDV (green). All results of a particular participant are depicted with the identical letter

The negative samples (sera 02/21 and 04/21, milk 09/21) were consistently correctly tested negative regardless of the applied test system, with exception of milk sample 09/21 which was tested “positive” in one participating laboratory (Table 2, Figs. 1 and 2). The overall specificity was 99.49% (95% confidence interval [CI] 97.20% to 99.99%). As the participant that tested the sample 09/21 positive did not indicate the unit in the results sheet, an evaluation as to whether the assessment was based on the instructions of the manufacturer and whether it is correct or false was not possible. The serum 01/21, which was taken subsequent to a BVDV-2 infection, tested consistently correctly positive (overall sensitivity for this sample 100.00%, 95% CI 94.94% to 100.00%). However, some discrepancies occurred when analyzing the other antibody-positive samples. The status of the serum 03/21, which originated from an animal immunized with an inactivated BVDV vaccine, was correctly identified as being positive by the neutralization test or by total antibody or E0-based ELISAs, while all applied NS3 (p80)-based ELISAs gave negative results (Table 2, Figs. 1 and 2). The overall sensitivity for this sample when using ELISA systems was 68.27% (95% CI 58.42% to 77.05%), with a sensitivity of 0.00% (95% CI 0.00% to 10.58%) for the NS3 (p80)-based ELISAs and 100.00% (95% CI 90.51% to 100.00%) for total antibody or E0-based ELISAs. The serum 05/21, which was taken after BDV infection, reacted positive in all ELISAs used. A differentiation between BVDV and BDV antibodies was only allowed by parallel application of neutralization tests against BVDV and BDV isolates. When BDV was not included in the virus panel against which the neutralization test was set up, the serum was assessed as BVDV antibody-positive (Fig. 2).

For the BVDV antibody-positive milk samples to be tested, there were in some cases considerable differences in the number of correct results, which depended (1) on the applied ELISA kit, and (2) on the used sample incubation protocol (Fig. 1). The milk samples 06/21 and 08/21, which represented an 1:1 mixture of antibody-positive and -negative individual milk samples (Table 1), have been tested 55 times. The sample 06/21 was tested negative by the participants two times (2/55, 3.6%; sensitivity 96.36%, 95% CI 87.47% to 99.56%) and the sample 8/21 was tested negative 31 times (31/55, 56.4%; sensitivity 43.64%, 95% CI 30.30% to 57.68%). For the milk sample 06/21, both false-negative results were produced by using a short sample incubation protocol (Fig. 1). In an additional case, the sample 06/21 was evaluated positive by one participant, although it did not exceed the cut-off as indicated in the instructions of the kit manufacturer (ELISA: Monoscreen AbELISA BVDV (NS3)/blocking). The incorrect negative results for the milk sample 08/21 were generated by either using a short or unknown incubation protocol (Fig. 1; ELISAs: ID Screen® BVD p80 Antibody Competition, BVDV p80 Ab Test, Svanovir® BVDV-Ab Confirmation) or by applying one of the following ELISA kits: Monoscreen AbELISA BVDV (NS3)/blocking (1/1, 100%), BVDV Total Ab Test (16/16, 100%), Svanovir® BVDV-Ab Screening (2/3, 66.7%), PrioCheck^TM^ Ruminant BVD p80 Ab Serum & Milk Kit (2/2, 100%).

Finally, the milk samples 07/21 and 10/21 simulated a herd prevalence of 20% by merging milk samples obtained from 2 seropositive animals with 8 seronegative milk samples (Table 1). The sample 07/21 tested negative 24 times (24/55, 43.6%; sensitivity 56.36%, 95% CI 42.32% to 69.70%), and the sample 10/21 in 28 cases (28/55, 50.9%; sensitivity 49.09%, 95% CI 35.35% to 62.93%). Again, the negative results were related to a short or unknown incubation period (ELISAs: ID Screen® BVD p80 Antibody Competition, BVDV p80 Ab Test, Svanovir® BVDV-Ab Confirmation) or the following ELISA kits (Fig. 1, Table 2): Monoscreen AbELISA BVDV (NS3)/blocking, BVDV Total Ab Test, Svanovir® BVDV-Ab Screening, PrioCheck^TM^ Ruminant BVD p80 Ab Serum & Milk Kit.

Overall, the pooled milk samples were only tested consistently positive when using the ID Screen® BVD p80 Antibody competition or the Svanovir® BVDV-Ab Confirmation ELISA in combination with the respective long sample incubation period (*n*= 16 and =2, respectively), or the PrioCheck^TM^ Bovine BVDV Ab Plate Kit (*n*=1).

## Discussion

Serological methods are a key component during the surveillance phase of BVDV control programs, when rising seroprevalences are indicative for a new introduction of one or more PI animals into a herd. Besides, serological methods might be applied during purchase investigation to identify so called “Trojan cows”, i.e. pregnant dams infected during the current gestation and therefore at risk for giving birth to a PI calf. In addition to PI calves, “Trojan cows” are a major cause for virus spread into BVDV-free herds [26–29, 47–49] and need to be identified as early as possible in order to separate them during the parturition period from further pregnant cows. Such “Trojan cows” represent a particular challenge when eliminating BVDV from a given area, as the problem becomes visible only after the birth of the PI calf, which might be several months after the purchase of the pregnant dam. Hence, for their early identification serological methods could be beneficial, provided they are applied regularly and sufficient sensitive tests are used.

In this interlaboratory comparison, individual sera obtained from BVDV-infected animals were generally correctly identified by every ELISA format. However, as reported previously [45, 50–55] also in this ring trial NS3-based ELISAs showed lower sensitivities for the serum sample obtained from an animal that has been immunized with an inactivated BVDV vaccine. NS3 is produced in large amounts during virus replication in infected animals or after immunization with live vaccines, thereby inducing the production of antibodies against this non-structural protein. In contrast, inactivated vaccines might contain mainly NS3-free BVD virions and do not replicate in the immunized animals. Therefore, the induction of an antibody response against NS3 relies only on the protein load already present in the vaccine [53]. Hence, sera from vaccinated animals might test negative in NS3-based assays, although high titers of antibodies directed against further proteins are measurable. Thus, for herds with animals vaccinated with inactivated vaccines, the application of total antibody or E^rns^-based ELISAs or of the neutralization test, which predominantly detects antibodies against the envelope glycoprotein E2, is recommended.

In terms of sample materials, milk is a convenient to collect and cost-effective alternative to serum, given that sampling of sera and analyses of herds by spot testing was the biggest cost driver during the transition from ear notch-based testing to serological surveillance in Switzerland [56]. When compared to the testing of individual sera, the investigation of bulk milk in dairy herds has the advantage that it can be performed more frequently at lower costs. However, lower sensitivities of commercial ELISAs have been reported for milk as sample matrix [46, 54, 57]. Therefore, bulk milk samples would most likely score only positive when a sufficient proportion of cows contributing to the pool seroconverted. This poorer diagnostic sensitivity could be at least partially decreased by routinely using the long incubation protocol of the ELISAs and by using the best performing test systems. As demonstrated in this proficiency trial and previously observed during a study comparing diverse commercial BVDV antibody ELISAs [45], the long incubation protocol often resulted in an increased diagnostic sensitivity compared to the short protocol of the respective test. Therefore, the standard application of a long-term incubation protocol is strongly recommended for the analysis of (bulk) milk samples. Nevertheless, independent of the used test and protocol, regular bulk milk analyses offer the possibility to compare current data to historical results, thereby identifying an increase (or decrease in case of competitive ELISAs) of the S/P% (S/N%) or % inhibition values, which might be indicative for a BVDV infection in the respective herd [58, 59].

It was previously reported that besides the incubation protocol and antigen used for ELISA plate coating, the ELISA format could influence the sensitivity of the respective test. An evaluation of 16 commercial antibody ELISAs suggested that competitive ELISAs show a lower diagnostic sensitivity than indirect tests also for milk samples [45]. Interestingly, the kits that performed best for pooled milk samples in this interlaboratory proficiency trial belong to both categories, as the best performance of all kits, which were used in more than one laboratory, for this sample matrix was achieved by the competitive ID Screen® BVD p80 Antibody Competition ELISA and by the indirect Svanovir® BVDV-Ab Confirmation ELISA, given that the respective long sample incubation protocol was applied.

In addition to the diagnostic sensitivity, the specificity is a key characteristic of diagnostic test systems. In the context of pestiviruses, the serological cross-reactivity of different virus species, e.g. between BVDV and BVD, represents unfortunately a major issue. As shown by the results of the serum sample 05/21, none of the currently applied ELISA test is able to differentiate anti-BVDV from anti-BDV antibodies. BVDV and BDV are closely related and both viruses may be transmitted between cattle and small ruminants, predominantly sheep, when those species are kept together [56, 60]. In Switzerland, for instance, up to 10% of all pestivirus antibody-positive cattle sera were reactive to BDV rather than to BVDV [61, 62]. However, disease control programs are generally restricted to BVDV in *Bovidae*. To correctly attribute antibodies to one of the virus species, thereby avoiding restrictions and costs for farms in case of BDV instead of BVDV infections, labor-intensive and costly neutralization assays using different virus strains, preferentially adjusted to the epidemiolocal situation in the respective area, are necessary [36].

## Conclusions

The presented interlaboratory proficiency trial for serological BVD diagnostics revealed, dependent on the test system and incubation period, considerable differences in the number of correct evaluations for BVDV-seropositive samples, most notably when considering the results obtained for pooled milk samples. Therefore, thorough validation and careful selection of the best performing ELISA tests is highly recommended, especially for laboratories analyzing samples in the context of the surveillance phase of eradication programs or in order to identify pregnant dams at risk for the birth of a PI calf. Here, in the context of an interlaboratory proficiency trial, the best performance for pooled milk samples of all kits, which were used in more than one laboratory, was achieved by the ID Screen® BVD p80 Antibody Competition and Svanovir® BVDV-Ab Confirmation ELISAs performed using the long sample incubation protocol.

## Methods

Five sera and five milk samples were sent to the participants, which were asked to investigate these samples with the methods and test systems routinely used in their laboratory. The sera comprised two cattle samples seronegative against pestiviruses (IDs 02/21 and 04/21), a sample taken from a cattle immunized with the inactivated BVDV-1 vaccine Bovilis® BVD-MD (MSD Tiergesundheit, Haar, Germany) (03/21), and sera obtained after experimental infection with BVDV-2 (01/21) or BDV (05/21), respectively. While all sera represented individual samples, the milk samples were prepared to mimic bulk tank milk with seroprevalences of 50% (06/21 and 08/21) and 20% (07/21 and 10/21), respectively. For that, four seropositive milk samples were merged with four negative milk samples or two seropositive with eight negative milk samples. The remaining milk sample (09/10) was seronegative UHT milk. With exception of the long-life milk, corresponding sera were available for all milk samples and they were tested for BVDV-specific antibodies by a standard microneutralization test [63] against BVDV-1 strain NADL. The resulting neutralizing titers are given in Table 1.

Aliquots of 1ml were prepared of all serum and milk samples in 2-ml injection bottles (Zscheile & Klinger GmbH, Hamburg, Germany). Thereafter, the samples were lyophilized and the injection bottles were sealed with rubber plug and flanged caps (Zscheile & Klinger GmbH, Hamburg, Germany). The aliquots were stored at 4°C until sent to the participating institutions.

The ring test sample panel was investigated by a total of 51 veterinary diagnostic laboratories from 15 countries (Austria, Belgium, Denmark, France, Germany, Ireland, Israel, Italy, Latvia, Lithuania, the Netherlands, Poland, Russia, Switzerland, United Kingdom) and three manufacturers of commercial ELISA kits. The following commercial antibody ELISA kits were used by the participating laboratories: Monoscreen AbELISA BVDV (E0)/blocking (E^rns^ (E0)-based; Bio-X Diagnostics S.A., Rochefort, Belgium), Monoscreen AbELISA BVDV (NS3)/blocking (NS3 (p80)-based; Bio-X Diagnostics S.A.), ID Screen® BVD p80 Antibody Competition (NS3 (p80)-based; Innovative Diagnostics, Grabels, France), BVDV Total Ab Test (configured by immobilizing BVDV antigen on the plates; IDEXX, Westbrook, United States), BVDV p80 Ab Test (NS3 (p80)-based; IDEXX), Svanovir® BVDV-Ab Screening (plates coated with non-infectious BVDV antigen; SVANOVA, Uppsala, Sweden), Svanovir® BVDV-Ab Confirmation (plates coated with non-infectious BVDV antigen; SVANOVA), PrioCheck^TM^ Ruminant BVD p80 Ab Serum & Milk Kit (NS3 (p80)-based; Thermo Fisher Scientific, Waltham, United States), PrioCheck^TM^ Bovine BVDV Ab Plate Kit (NS3 (p80)-based; Thermo Fisher Scientific). In one laboratory, an in-house ELISA was applied. For some of the commercial kits two distinct sample incubation protocols are proposed in the kit manual (short incubation protocol: kit-dependent 1 or 2 hours; long incubation protocol: kit-dependent 12 up to 20 hours). Both protocols were applied by the participants. Furthermore, the sera were tested in 28 laboratories by the cell-culture based standard microneutralization test against diverse BVDV-1, BVDV-2 and/or BDV strains.

The sensitivities and specificities as mentioned in the Results section were calculated by using the free statistical calculator MedCalc (MedCalc Software, Ostend, Belgium).

## Data Availability

The datasets supporting the conclusions of this article are included within the article.

## Abbreviations

BDV: border disease virus
BVD: bovine viral diarrhea
BVDV: bovine viral diarrhea virus
cp: cytopathic
CSFV: classical swine fever virus
FCS: fetal calf serum
ncp: non-cytopathic
PI: persistently infected
